# Genome-wide analysis of haloacid dehalogenase genes reveals their function in phosphate starvation responses in rice

**DOI:** 10.1371/journal.pone.0245600

**Published:** 2021-01-22

**Authors:** Zezhen Du, Suren Deng, Zixuan Wu, Chuang Wang

**Affiliations:** 1 Microelement Research Center, College of Resources & Environment, Huazhong Agricultural University, Wuhan, P. R. China; 2 Key Laboratory of Arable Land Conservation (Middle and Lower Reaches of Yangtze River), MOA, Huazhong Agricultural University, Wuhan, P. R. China; Weizmann Institute of Science, ISRAEL

## Abstract

The HAD superfamily is named after the halogenated acid dehalogenase found in bacteria, which hydrolyses a diverse range of organic phosphate substrates. Although certain studies have shown the involvement of HAD genes in Pi starvation responses, systematic classification and bioinformatics analysis of the HAD superfamily in plants is lacking. In this study, 41 and 40 HAD genes were identified by genomic searching in rice and *Arabidopsis*, respectively. According to sequence similarity, these proteins are divided into three major groups and seven subgroups. Conserved motif analysis indicates that the majority of the identified HAD proteins contain phosphatase domains. A further structural analysis showed that HAD proteins have four conserved motifs and specified cap domains. Fewer HAD genes show collinearity relationships in both rice and *Arabidopsis*, which is consistent with the large variations in the HAD genes. Among the 41 HAD genes of rice, the promoters of 28 genes contain Pi-responsive cis-elements. Mining of transcriptome data and qRT-PCR results showed that at least the expression of 17 HAD genes was induced by Pi starvation in shoots or roots. These HAD proteins are predicted to be involved in intracellular or extracellular Po recycling under Pi stress conditions in plants.

## Introduction

Phosphorus (P) is one of the essential macronutrients for plant growth and development. There are two different forms of P in soil: inorganic P (Pi) and organic P (Po), of which plants can only absorb and utilize water-soluble Pi. Although P is abundant in the Earth’s crust, it is usually present in soil in the form of Po or fixed with other metals, making it unavailable to plants and insufficient to support the optimal growth of plants [1, 2]. To cope with Pi deficiency stress, plants have evolved a series of physiological and biochemical strategies to enhance the uptake and utilization of P [3]. Inducing the expression and synthesis of phosphatases is one of the important responses to Pi stress in plants [4].

Phosphatase (EC 3.1.3) is a type of hydrolase that acts to free attached phosphate groups from other molecules [5]. It was reported that Pi starvation induced the synthesis of both intracellular and extracellular acid phosphatase isozymes in plants [6]. More specifically, the activity of intracellular acid phosphatases (IAPs) increases after two days of Pi starvation, which is considered to be involved in recycling Pi from intracellular Po compounds [6, 7]. In contrast, the activity of secreted acid phosphatases (SAPs) is enhanced under prolonged Pi starvation conditions and participates in the degradation of extracellular Po [6, 8, 9]. Many Pi starvation-induced IAPs and SAPs have been purified from different plant species, and the majority of them are encoded by purple acid phosphatase (PAP) genes [8, 10–16]. Compared with PAP genes, other types of Pi starvation-induced phosphatases are less studied in plants.

The HAD superfamily is named after the halogenated acid dehalogenase in bacteria, which catalyses carbon or phosphoryl group transfer reactions on a diverse range of substrates [17]. Enzymes exist in all three superkingdoms of life, showing very disparate biological functions [18]. The majority of HAD proteins are involved in phosphoryl transfer, including phosphatases, P-type ATPases, phosphonatases, and phosphotransferases [19]. All proteins of the HAD superfamily contain a similar core catalytic domain, which is a unified Rossmannoid fold [18]. Moreover, members of the HAD superfamily insert three kinds of cap structures in the core Rossmannoid fold [18]. Although the HAD superfamily proteins show little overall sequence similarity (<15%), these proteins contain four highly conserved sequence motifs [19, 20].

Several HAD genes have been reported to regulate Pi deficiency stress responses in different plant species. In *Arabidopsis*, the expression of *AtPECP1* and *AtPS2* was significantly induced by Pi starvation. Subsequently, these enzymes were proven to recycle Pi from phosphocholine and phosphoethanolamine under Pi starvation conditions [21–25]. Pi starvation also induces the secretion of HAD phosphatases in *Arabidopsis*, which may participate in the utilization of extracellular Po [26]. However, mutation or overexpression of these HAD genes does not show any growth phenotypes under either Pi-replete or Pi-depleted conditions in *Arabidopsis* [23, 26]. In contrast, overexpression of *OsHAD1* significantly enhanced extracellular Po utilization and stimulated the growth of rice under Pi-deficient conditions [27]. Overexpression of *LePS2* resulted in increased acid phosphatase activity and anthocyanin accumulation and delayed flowering in tomatoes [28]. Similarly, Pi starvation-induced *PvPS2*.*1* and *PvPS2*.*2* increase the Pi adaptation ability in plants [29]. Moreover, soybean *GmHAD1* was identified to be associated with tolerance to low Pi stress by map-based cloning, and *GmHAD1* overexpression led to increased acid phosphatase activity and P utilization efficiency in both soybean and *Arabidopsis* plants [30].

Although a number of HAD genes are involved in Pi starvation responses in plants, systematic classification of the HAD superfamily in plants and functional prediction of Pi stress adaptation are lacking. Because the protein similarities of HAD family members are relatively few, it is difficult to search out all HAD family genes by direct blastP as PAPs [4]. Therefore, the hidden Markov model (HMM) of the HAD family was built and used to search against the rice and *Arabidopsis* protein sequences. Forty-one and 40 typical HAD proteins have been identified in rice and *Arabidopsis*, respectively. A further combination of bioinformatics and expression analysis showed that many HAD genes may be involved in intracellular and extracellular Po degradation under Pi stress conditions in plants.

## Materials and methods

### Identification of HAD proteins and motifs in rice and *Arabidopsis*

The whole protein sequences of *Arabidopsis* thaliana and rice were downloaded from the Ensembl database (http://plants.ensembl.org). Hidden Markov models (HMMs) of the HAD family were obtained from the Pfam protein database (http://pfam.xfam.org/) and constructed from reported plant HAD proteins [31]. The two hidden Markov models of the HAD domain were used to search against the *Arabidopsis* and rice protein sequence data by HMMER software using default parameters (E-value = 0.5) [32]. The identified proteins were further examined by hand to verify whether the protein contained conserved motif I of HAD proteins. The final sequences were defined as HAD proteins and submitted to ExPASy (https://web.expasy.org) to calculate the isoelectric point and molecular weight [33]. Conserved motifs of the identified proteins were predicted by MEME using the following parameters: the search window of motifs was between 2–30 amino acids, and the motif number was set as 20 [34].

### Chromosomal location, gene structure, and synteny analysis

The corresponding gene loci of the identified proteins were downloaded from the Ensembl database (http://plants.ensembl.org). All the gene loci were mapped on the *Arabidopsis* and rice chromosomes with Mapchart. Gene collinearity relationships were analysed by MCScanX [35, 36]. The collinear relationship of HAD genes from *Arabidopsis* and rice was finally drawn by Circos [35]. Introns and exons of all the gene loci were extracted from the rice comment files of the Ensembl database and drawn with TBtools [37].

### Phylogenetic analysis

The full-length protein sequences of rice and *Arabidopsis* were aligned by MEGA-X using default parameters. The unrooted maximum likelihood method phylogenetic tree was constructed by MEGA-X using the Jones-Taylor-Thornton (JTT) model [38].

### Analysis of cis-elements in the promoters of HAD genes

The promoter sequences (1.5 Kb) of rice HAD genes were downloaded from the Ensembl database (http://plants.ensembl.org) and were then submitted to the PlantCARE database for cis-element analysis [39]. The positions of cis-element *P1BS* and the W-box are marked on the promoters.

### Protein three-dimensional structure simulation

Three-dimensional structures of the HAD proteins were built by SWISS-MODEL (https://swissmodel.expasy.org/) [40]. Targeted proteins were imported, and the template was searched by the software. Homologous modelling was used to simulate the 3D structure based on the template. Each model with a sequence similarity of greater than 30% was employed for the next step.

### Plant materials and growth condition

Rice seeds (Oryza sativa, cv Nipponbare) were sterilized with 1% nitric acid and then germinated at 24°C in the dark for two days. Uniform germinated seeds were grown in normal nutrient solution for 10 days and then transferred to nutrient solutions without Pi for 10 days, followed by two days of recovery in normal solution. The nutrient solution contained 1.425 mM NH_4_NO_3_, 0.998 mM CaCl_2_·2H_2_O, 0.323 mM NaH_2_PO_4_·2H_2_O, 0.513 mM K_2_SO_4_, 1.643 mM MgSO_4_·7H_2_O, 9.5 μM MnCl_2_·4H_2_O, 0.075 μM (NH_4_)_6_Mo_7_O_24_·4H_2_O, 19 μM H_3_BO_3_, 0.152 μM ZnSO_4_·7H_2_O, 0.155 μM CuSO_4_·5H_2_O, 0.125 mM FeSO_4_·7H_2_O, 0.125 mM Na_2_EDTA·2H_2_O, and 0.25 mM Na_2_SiO_3_·9H_2_O, pH 5.5. The hydroponic experiments were carried out in a greenhouse with a 12/12-h photoperiod (200 μmol photons m^–2^ s^–1^) at 30/24°C and 60% relative humidity. The solution was refreshed every three d.

### Quantitative real-time PCR analysis

Total RNA was extracted from the shoots or roots of rice plants using TRIzol reagent (CoWin). Two micrograms of RNA were used to synthesize cDNA using a reverse transcription kit (CoWin) according to the manufacturer’s instructions. qRT-PCR experiments were performed on SYBR Green Real-Time PCR Master Mix reagent (YEASEN) dye on an Applied Biosystems Quantstudio^TM^ Real-Time PCR System machine. The relative expression levels were calculated using the 2 –^ΔΔCT^ method [41]. The primer sequences of the qRT-PCR are listed in S3 Table.

### Statistical analysis

SPSS statistics base software (version 22) was used for statistical analysis. Significant differences were evaluated using one-way ANOVA and Tukey’s test.

## Results

### Identification of HAD genes in rice and *Arabidopsis*

To obtain the HAD proteins in rice and *Arabidopsis*, hidden Markov models of HAD were obtained from the Pfam protein database (PF12710) and constructed from reported plant HAD proteins (S1 Fig). The two different models of the HAD domain were used to search the whole protein sequence data of rice and *Arabidopsis* by HMMER [32]. Seventy-nine and 85 putative HAD proteins, which corresponded to 63 and 60 gene loci, were identified in rice and *Arabidopsis*, respectively (S2 Fig). Although the organization of HAD proteins is variable, the reactions catalysed by HAD require two core Asp residues in a DxD signature of motif I [42]. Then, only the sequences that contain the corresponding motif I are defined as typical plant HAD proteins (S2 Fig). In this way, 41 and 40 HAD genes were annotated as HAD coding genes in rice and *Arabidopsis*, respectively (S1 Table). The isoelectric points (pI) and molecular weight (MW) of the HAD proteins ranged from 4.65 to 10.78 and 19.7 to 117.7 kDa, respectively (S1 Table and S3 Fig).

### Phylogenetic and conserved motif analysis of the HAD proteins

**Fig 1:**
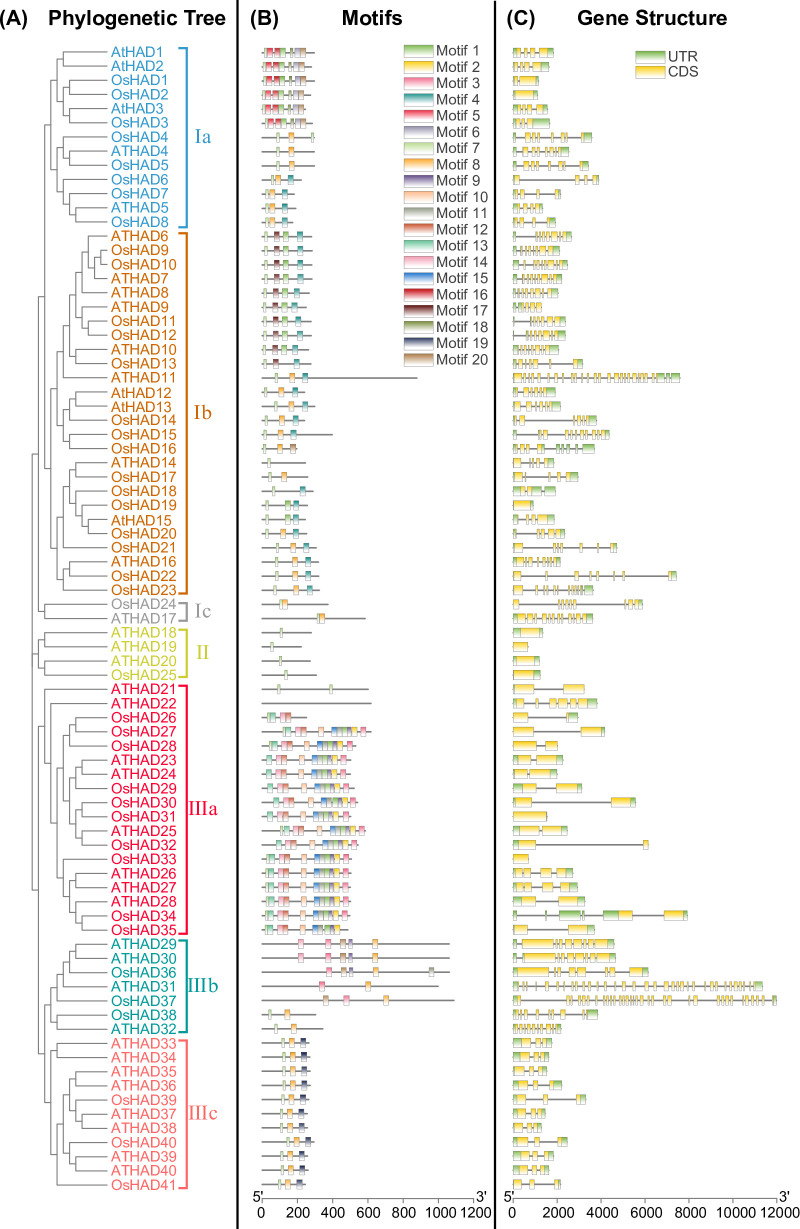
Phylogenetic tree (A), conserved protein motifs (B), and gene structures (C) of HAD genes from rice and *Arabidopsis*. The phylogenetic tree of the HAD protein family in rice and *Arabidopsis* was constructed using MEGA-X software based on the maximum likelihood method and a JTT matrix-based model. Different colours represent different subgroups. The conserved motifs of the protein were analysed with MEME software and marked with different colours. For the gene structures, green represents untranslated 5′- and 3′-regions, and yellow represents exons.

To study the evolutionary relationship between HAD family genes, the identified rice and *Arabidopsis* HAD protein sequences were aligned, and a phylogenetic tree was constructed by the maximum likelihood method. The 81 proteins were divided into three major groups (families I, II, and III) and seven subgroups (subfamilies Ia, Ib, Ic, II, IIIa, IIIb, and IIIc), each with more than 95% bootstrap support (Fig 1A). The rice and *Arabidopsis* HAD genes are named according to the phylogenetic tree from Ia to IIIc, which are *OsHAD1* to *OsHAD41* and *AtHAD1* to *AtHAD40*, respectively (Fig 1A and S1 Table).

The motif variation of HAD proteins was further analysed by MEME and annotated with the Pfam database. Twenty motifs with the lowest E-values are listed (Table 1). Interestingly, five motifs (motifs 4, 7, 8, 12, and 19) were annotated as haloacid dehalogenase-like hydrolases or HAD superfamilies, while six other motifs (motifs 1, 5, 6, 16, 18 and 20) were annotated as phosphatases (Table 1). The functions of motifs 2, 3, 9, 10, 11, 13, and 17 are unknown. These 20 motifs were then mapped on each HAD protein. HAD proteins from the same subgroups have a similar motif arrangement, which supported the results of phylogenetic analysis (Fig 1B). Moreover, the number of conserved motifs ranges from one to 11 in different HAD members, which is consistent with the large divergence of HAD proteins.

**Table 1:** Information of the 20 motifs in HAD proteins.

Motif	Sequence	Annotation
Motif-1	SBABAAFIERILKHLGLVDCFEGITCREPY	Putative Phosphatase
Motif -2	MFHGTTARGWKGLDPYFFFMNPRPAYEVTF	-
Motif -3	NYVQRVJASVLGFECTTLTRKDKYRVLAGN	-
Motif -4	GVBPKKTLFFEDSVRNIQAGKAAGMHTVLV	Haloacid dehalogenase-like hydrolase
Motif -5	NWVVDELGFTDLFNQLLPTMPWNSLMDRMM	Putative Phosphatase
Motif -6	YLGDGSGDYCPSLKLKEKDYMMPRKNFPVW	Putative Phosphatase
Motif -7	VDVVVFDLDGTLLDS	haloacid dehalogenase-like hydrolase
Motif -8	KAPLRPGVLKLYKELKEKGIKVALASGRN	haloacid dehalogenase-like hydrolase
Motif -9	LLRFSALFAELTDRIVPVAMB	-
Motif -10	PVPRERLPKPVIFHDGRLVQRPTPLAALL	-
Motif -11	GRPITAVTYSISRLSEIJSPIPTVRLTRD	-
Motif -12	RRVVVTASPRVMVEPFLKEYLGADKVVGT	haloacid dehalogenase-like hydrolase
Motif -13	SAFPYFMLVAFEAGGLLRALLLLLLAPFVW	-
Motif -14	RDVEAVARAVLPKFYAADVHPDAWRVFASC	Sarcosine oxidase, gamma subunit family
Motif -15	PPPPSPGRPGVLFVCNHRTLLDPVVLATAL	Acyltransferase
Motif -16	DIEZVLRRIPLHPRVIPAIKAAHALGCDLR	Putative Phosphatase
Motif -17	TTMAGLKALGYEFDYDEYHSYVHGRLPYE	-
Motif -18	CPPNMCKGLIIERIQD	Putative Phosphatase
Motif -19	YKSEKRKELVKEGYRIRGNSGDQWSDLLGF	HAD superfamily (Acid phosphatase)
Motif -20	LICKBPSLVKAEVHEWSDGEELEQVLLHLI	Putative Phosphatase

**Fig 2:**
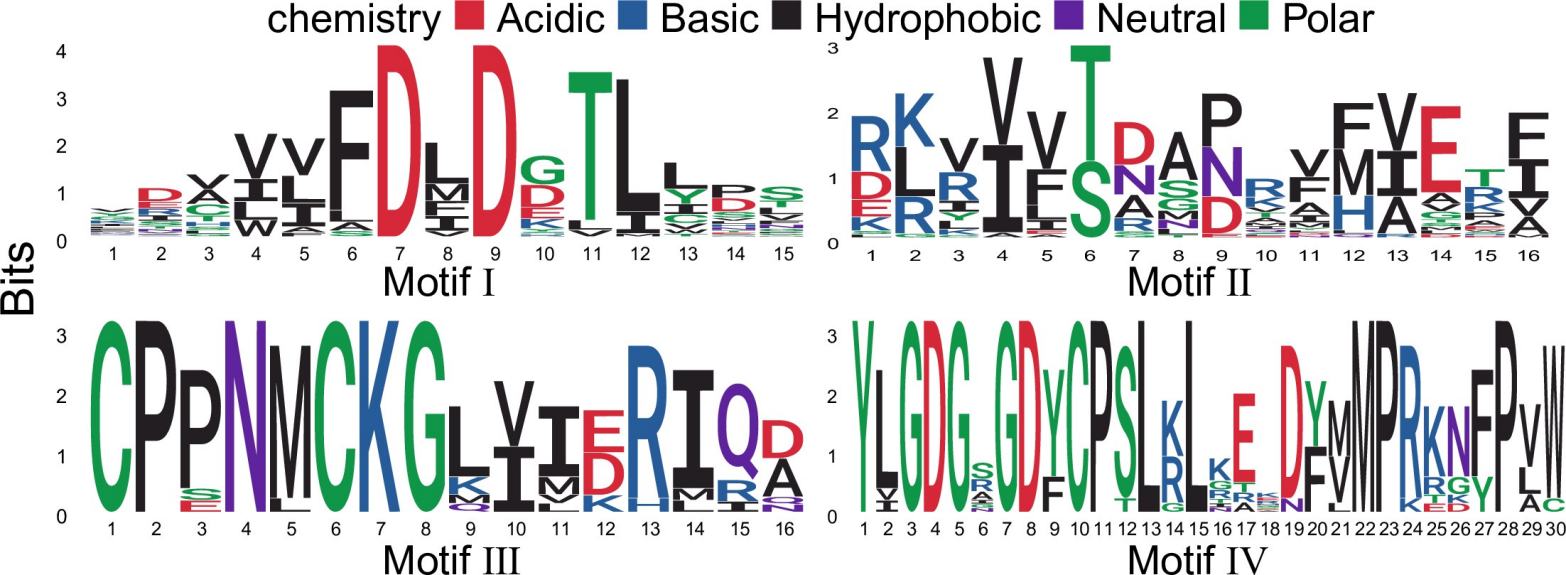
Graphical sequence logo representation of the four conserved motifs in plant HAD proteins. Signatures of the motif were motif I: DXD; motif II: S/T; motif III: K; motif IV: GDXXXD.

Although the overall homology between HAD groups is very low, sequence comparisons have shown that all members of the HAD proteins contain four conserved motifs (Fig 2), which could be used to define HAD proteins in plants. To determine whether the HAD proteins are secreted, the signal peptides and subcellular localization were predicted by using TargetP 2.0. In total, only 11 HAD proteins contained a signal peptide in rice and *Arabidopsis* (S2 Table).

### Cap modules of HAD family in plants

In addition to the different conserved motifs, the HAD proteins were classified by their structure. Three different cap modules (C0, C1, and C2), which determine the substrate specificity, have been found in the HAD superfamily. There were 2, 34, and 5 HAD proteins in rice with C0, C1, and C2 caps, respectively (S1 Table). Similarly, 2 C0, 35 C1, and 3 C2 caps were found among the HAD proteins in *Arabidopsis* (S1 Table).

**Fig 3:**
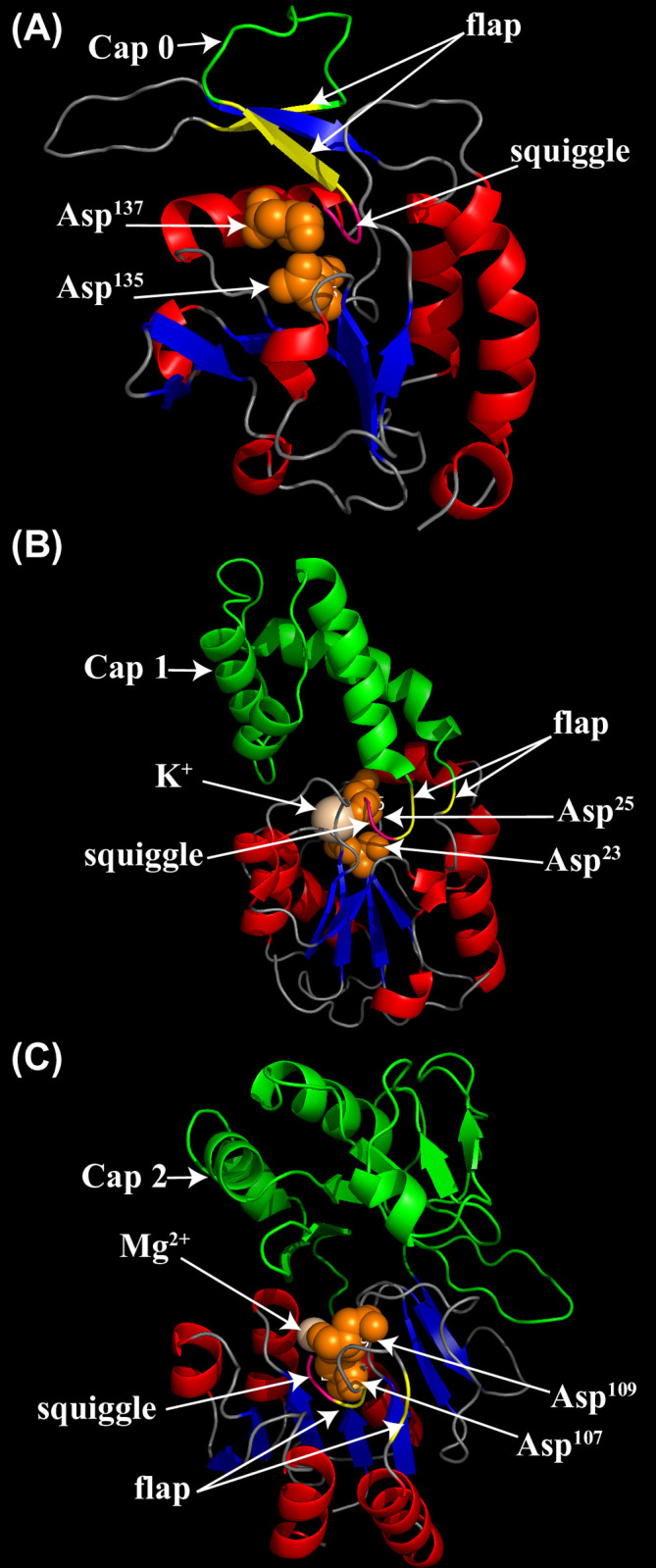
Simulated three-dimensional structures of typical HAD proteins in rice by SWISS-MODEL. (A) The three-dimensional structure of the OsHAD25 protein, which represents the C0 cap domain. The template accession No. is 3 pgl.1. A for OsHAD25. (B) The three-dimensional structure of the OsHAD14 protein, which represents the C1 cap domain. The template accession No. is 3 l5k.1. A for OsHAD14. (C) The three-dimensional structure of the OsHAD24 protein, which represents the C2 cap domain. The template accession No. is 1rkq.1. A for OsHAD24. The β strands are coloured blue, while the α helices are coloured red. The special flap and squiggle structures are coloured pink and yellow, respectively. Cap domains are marked in green. Two conserved aspartic acid residues of motif I are marked orange.

Three-dimensional structures of *OsHAD14*, *OsHAD24*, and *OsHAD25*, which represent proteins of the C0, C1, and C2 caps, were simulated by SWISS-MODEL. As expected, all three types of proteins have a squiggle and a flap structure in their core catalytic domain, which are two key structural signatures of the HAD domain (Fig 3). The C0 cap of *OsHAD25* has a small insertion between the two strands of the flap, while the C1 cap of *OsHAD14* contains four alpha-helices in the middle of the flap motif (Fig 3A and 3B). In contrast, the C2 cap has a more complex insertion that contains four α-helices and five β-sheets (Fig 3C).

### Gene structure, chromosomal localization and collinearity analysis of HAD genes in rice and *Arabidopsis*

Genomic sequences of the HAD genes were downloaded from the database and used to analyse the gene structures. There were great differences in the gene structures of the HAD genes, and the number of introns ranged from 0 to 33 (Fig 1C). Moreover, the HAD genes were unequally distributed on the chromosomes of rice and *Arabidopsis* (S4 Fig).

**Fig 4:**
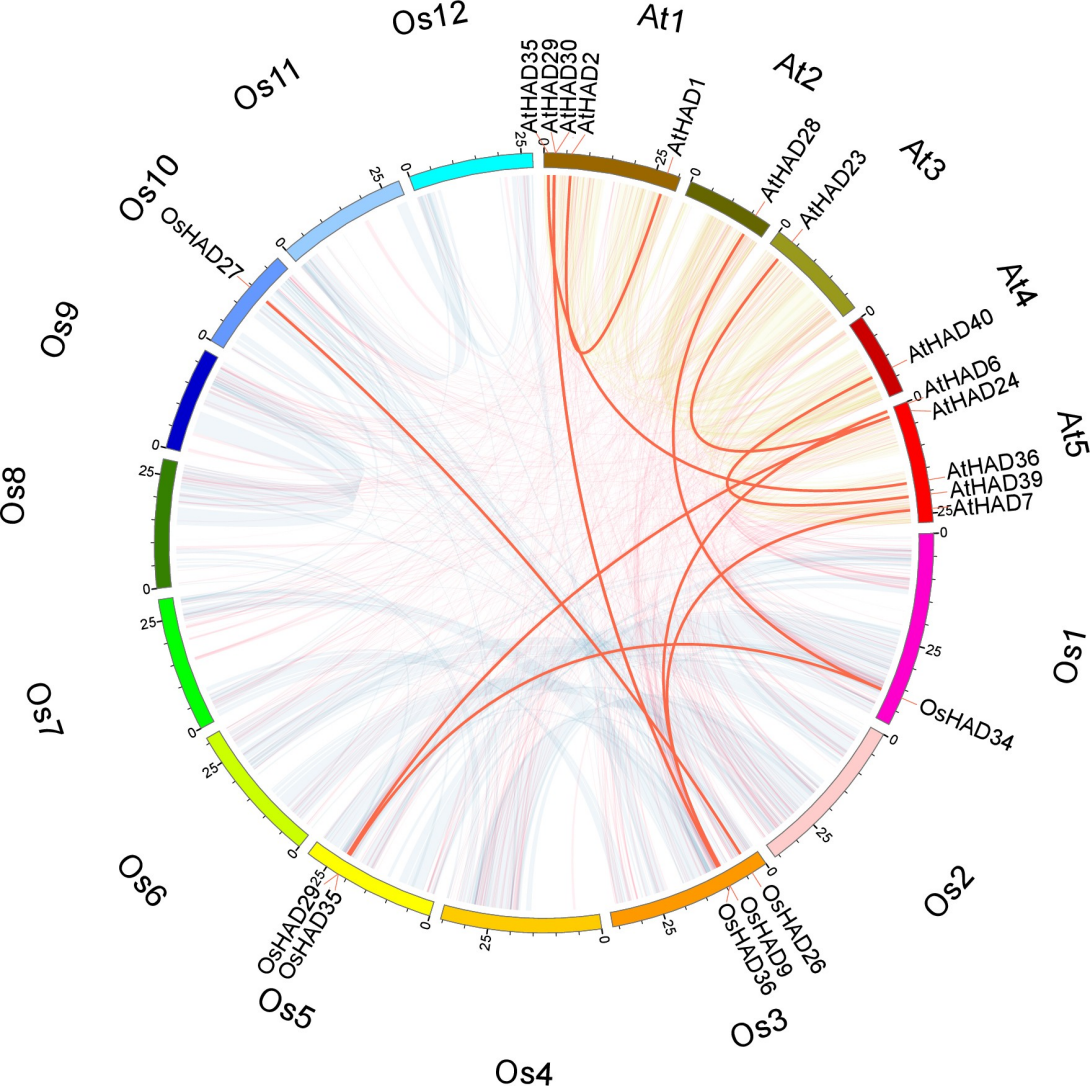
Chromosomal distribution and interchromosomal relationship between *HAD* family genes in rice and *Arabidopsis*. The transparent lines indicate all the collinear blocks in the rice and *Arabidopsis* genomes. The red lines indicate duplicated HAD gene pairs. The chromosome names are marked on the outermost edge of the circle.

To explore the collinearity relationship of genes between species in the evolutionary process, BLASTP and MCScanX software were used to analyse the HAD genes of *Arabidopsis* and rice, and the results were visualized with Circos. Only two and four pairs of collinearity relationships between HAD genes in rice and *Arabidopsis*, respectively, were observed (Fig 4). There were another six pairs of collinearity between HAD genes of rice and *Arabidopsis* (Fig 4). The low collinearity of HAD genes indicates that large variations exist among the HAD genes.

### Pi-responsive cis-acting elements and the expression of HAD genes under different Pi supply conditions

**Fig 5:**
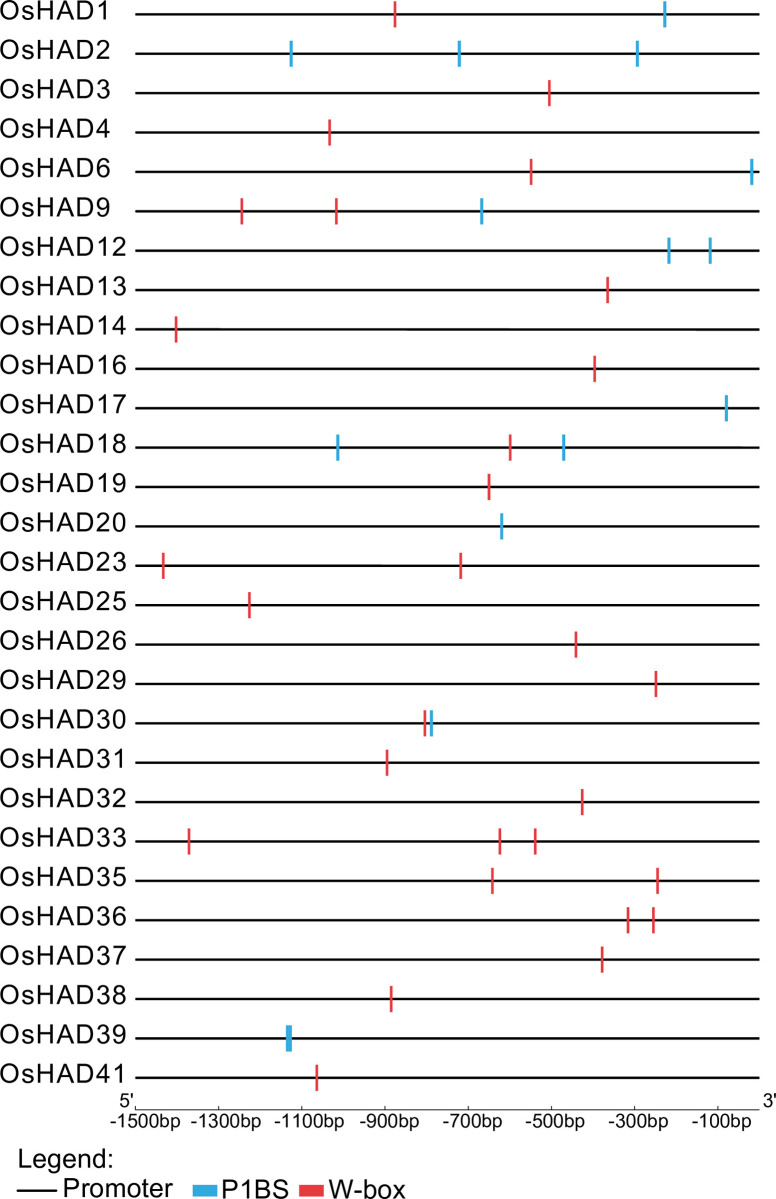
Distribution of the cis-element *P1BS* and W-box in the 1.5-kb regions upstream of ATG in the *HAD* genes of rice.

To explore whether the expression of HAD genes is regulated by Pi nutrients, a 1.5-kb promoter sequence was extracted to analyse cis-acting regulatory elements in rice. The promoters of 10 HAD genes contain the *P1BS* site (Fig 5), which is the binding site of PHR and PHR-like transcription factors [43]. Moreover, 23 promoters of HAD genes contain W-box sites (Fig 5), which are the binding sites of WRKY transcription factors [44]. Both the PHR and WRKY transcription factors are the key regulators of Pi starvation signalling in rice, indicating the putative relationship between HAD genes and Pi nutrients.

**Fig 6:**
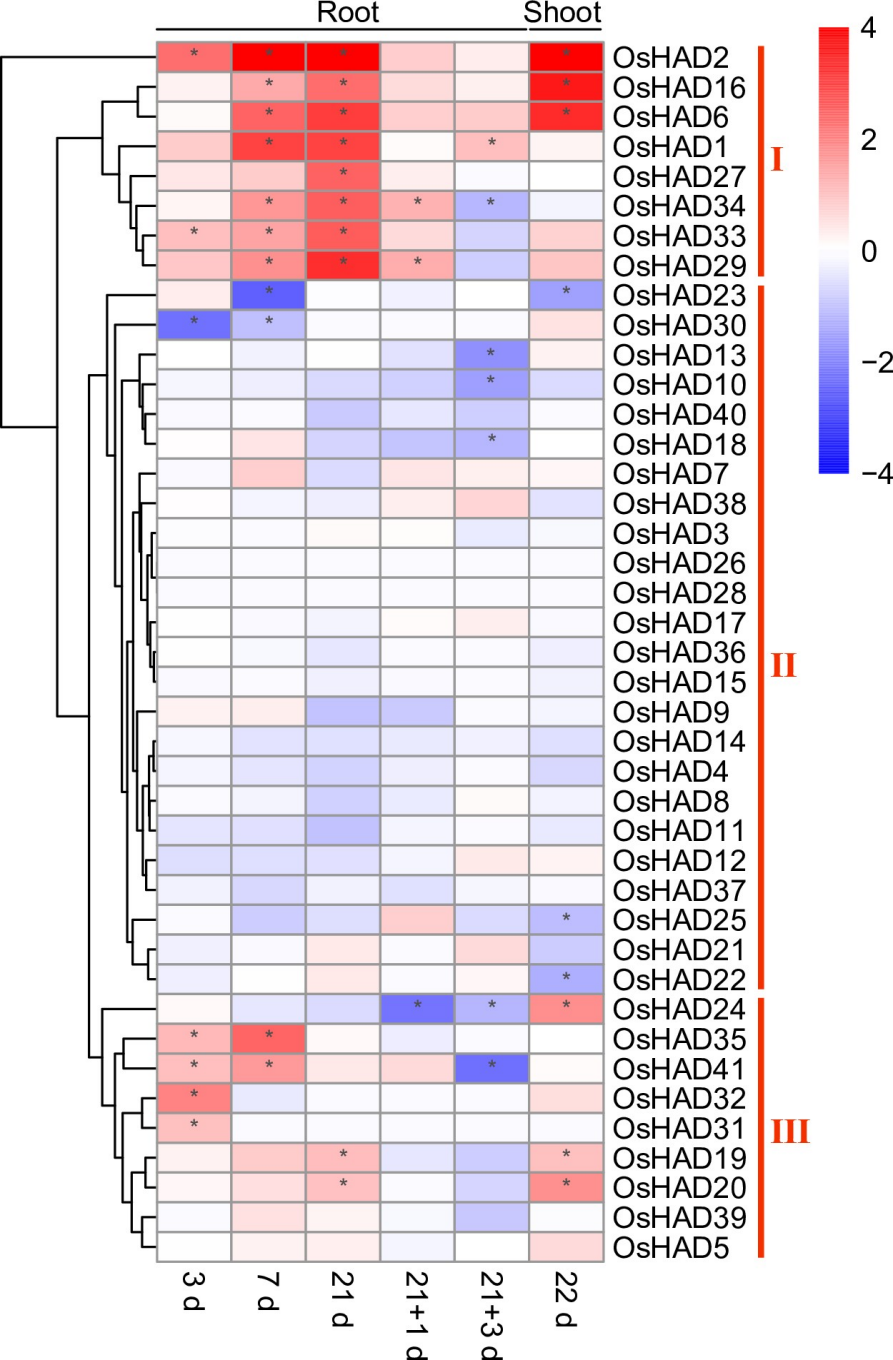
The expression profiles of *HAD* genes in roots and shoots of rice under Pi deficiency conditions. The RNA-seq data were downloaded from the DRYAD database (https://datadryad.org). 3d, 7d, 21d and 22d represent the seedlings that were treated under Pi-deficient conditions for 3, 7, 21 and 22 days. 21+1d and 21+3d represent the seedlings that were treated under Pi-deficient conditions for 21 days, followed by 1 and 3 days of recovery under normal conditions.

To further confirm whether the expression of HAD genes was regulated by Pi starvation, previously reported transcriptome data were reanalysed in rice [45]. The response of HAD genes to Pi nutrients can be divided into three different clusters (Fig 6). The first cluster contained eight genes, the expression of which showed a huge response to prolonged Pi starvation in roots. The re-supply of Pi for one day quickly recovered the Pi starvation-induced expression of these genes in roots. The expression of *OsHAD2*, *OsHAD6* and *OsHAD16* was also induced by Pi starvation by more than 10-fold in shoots (Fig 6). The expression of the second cluster of HAD genes did not respond to, or were suppressed by, Pi starvation conditions according to the transcript profiling. The third cluster of HAD genes showed a medium response to Pi starvation, while the expression of seven genes was induced by more than 2-fold under certain Pi starvation conditions in roots or shoots (Fig 6).

### qRT-PCR analysis of the transcripts of HAD genes under different Pi treatment conditions

**Fig 7:**
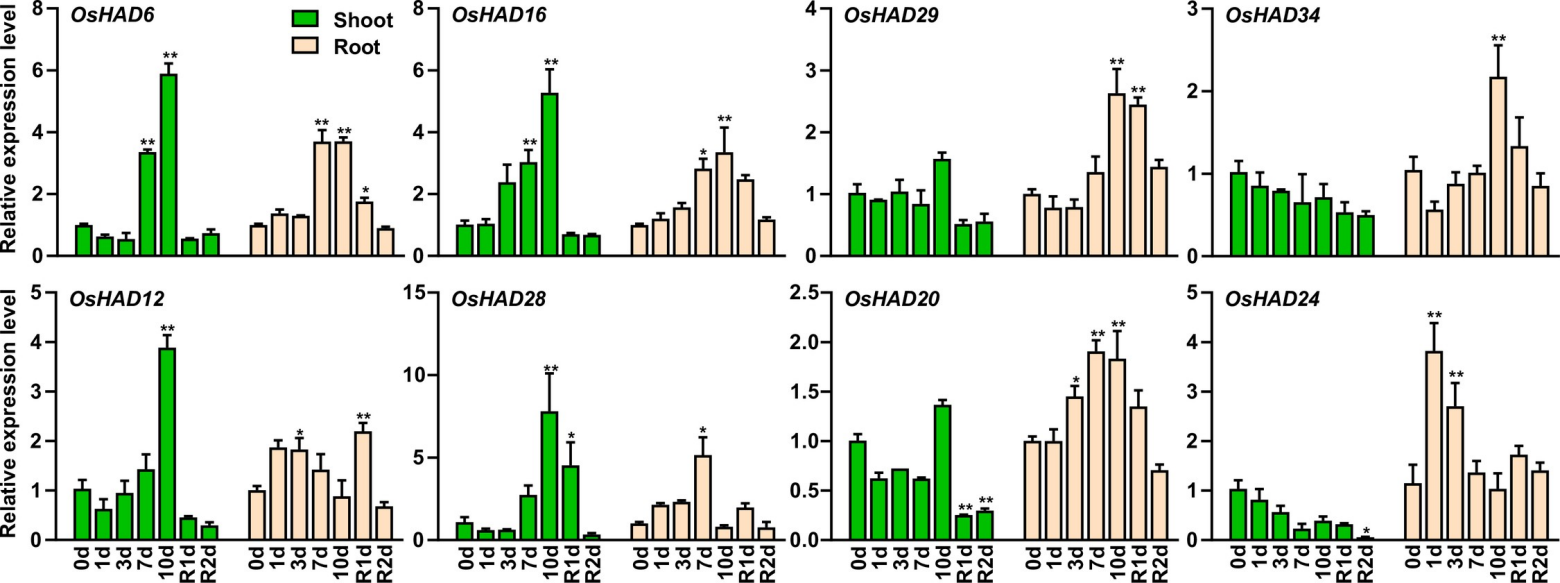
qRT-PCR analysis of the selected *HAD* genes in rice during a period of P starvation followed by resupply. Germinated seeds were grown in normal nutrient solution for 10 d and then transferred to a solution without Pi for 10 d, followed by 2 d recovery (R) in normal solution. RNA was extracted for quantitative RT-PCR, and the expression of *HAD* genes was normalized to that of *OsACTIN*. Data are means (±SEM) of three replicates. Significant differences compared with the control (0 d) were determined using Tukey’s test (*P<0.05; **P<0.01).

Eight HAD genes were further selected, and their expression was measured by qRT-PCR under different Pi conditions. Among the eight selected *HAD* genes, *OsHAD6*, *OsHAD16*, *OsHAD29*, and *OsHAD34* belong to cluster I genes, *OsHAD12* and *OsHAD28* belong to cluster II genes, and *OsHAD20* and *OsHAD24* belong to cluster III genes (Fig 6). Consistent with the transcription data, the expression of *OsHAD6*, *OsHAD16*, *OsHAD29*, and *OsHAD34* was significantly induced by prolonged Pi starvation in roots, while the expression of *OsHAD6* and *OsHAD16* was also induced by Pi starvation in shoots (Fig 7). The expression of *OsHAD20* and *OsHAD24* was induced by Pi starvation in roots and suppressed in the shoots by resupply Pi to Pi-starved plants (Fig 7). Although the transcription data showed that the transcripts of *OsHAD12* and *OsHAD28* did not respond to Pi starvation, a previous study detected the induced expression of *OsHAD12* under Pi starvation conditions [27]. qRT-PCR proved that the expression of *OsHAD12* and *OsHAD28* was induced by Pi starvation in both roots and shoots (Fig 7).

## Discussion

### Identification and structural analysis of HAD proteins in plants

HAD proteins are composed of ATPase (~ 20%) and acid phosphatase (~ 79%), which exist in almost all organisms [46]. There are 28 and 183 HAD proteins from *E*. *coli* and humans, respectively [46]. Although a number of HAD domain proteins have been reported, no research has systemically analysed HAD proteins at the genomic level in plants. According to the hydrolysing substrates of HAD proteins, these enzymes can be divided into protein phosphatases, small molecule phosphatases, dehalogenases, phosphonatases and β-phosphoglucose mutases [47]. Although sequence comparisons found that the HAD domain contains four conserved sequence motifs, the similarities of whole sequences of HAD superfamily members are relatively low (<15%) [48]. Therefore, it is impossible to search out all the HAD family genes by the BLAST tool. In this study, the proteins containing the HAD domain were searched using hidden Markov models of HAD (S1 and S2 Figs). Among the conserved motifs of HAD proteins, a mutation of aspartic acid in motif I would lead to a significant reduction or even loss of enzyme activity [42]. Therefore, the identified proteins by HMMER software were further screened with motif I, which finally found 41 and 40 HAD genes from rice and *Arabidopsis*, respectively (S1 Table).

Using the identified HAD proteins from rice and *Arabidopsis*, HMM of the four conserved motifs was extracted with typical signatures. There are two conserved aspartic acids (D) in both motifs I and IV, which are involved in coordinating Mg^2+^ in the active site (Fig 2). Motif II is characterized by a highly conserved serine or threonine (S/T), while motif III contains a conserved lysine (K) (Fig 2). Motifs II and III contribute to the stability of the reaction intermediates of the hydrolysis reaction [18]. In addition to the identified HAD proteins in this study, many other proteins were also annotated as HAD domain-containing proteins in the UniProt database. However, most of them lack critical motif I and were not analysed in this study.

Except for the four conserved motifs, the HAD superfamily shares common three-dimensional structures [48]. All HAD domains have a squiggle and flap in the Rossmannoid fold, which is distinguished from the Rossmannoid fold in other types of phosphatases [18]. Moreover, different kinds of cap structures insert in the middle of the flap structure or adjacent to motif III. The cap structures surround the catalytic centre and function to adjust substrate specificity [49, 50]. Therefore, understanding the cap structure is very important for studying the substrate specificity of the HAD protein. Among the 41 HAD genes in rice, two are C0 genes, five are C2 genes, and the rest are C1 cap structural genes (S1 Table). The cap structure has a great influence on the substrate specificity of the enzyme. Mutation of the amino acids (△F53, △N54, △N55) in the cap structure will reduce the kinetics of *AtHAD15* [51]. The substrate specificity of HAD proteins with C1 and C2 cap structures is lower than that of C0, which may be related to the specific enzyme residues of caps that can bind to different substrates [50].

### A number of HAD phosphatases are involved in intracellular and extracellular Po recycling

Eighty-one HAD proteins were found in this research and divided into seven subgroups (Ia, Ib, Ic, II, IIIa, IIIb, and IIIc). Among the 20 most conserved motifs in HAD proteins of plants, six are annotated as putative phosphatases, which is in accordance with the phosphoryl transfer activity of the HAD domain (Table 1). Interestingly, the promoters of 28 HAD genes contain *P1BS* or W-box in rice, indicating that these HAD genes may be regulated by Pi starvation (Fig 5). Combining transcriptome data and qRT-PCR analysis showed that the expression of at least 17 HAD genes was induced by Pi starvation in shoots or roots (Figs 6 and 7). These Pi-responsive HAD genes may participate in the regulation of Pi stress adaptation responses in rice.

It has been reported that plants synthesize intercellular and extracellular phosphatases to recycle different Po under Pi starvation conditions [16]. The majority of the Pi starvation-induced secreted acid phosphatases in plants are PAPs, which belong to a unique acid phosphatase subfamily. Interestingly, *AtVPS3* (*AtHAD37*) was recently identified as a novel secreted acid phosphatase isoform under Pi starvation conditions in *Arabidopsis* [26]. Moreover, *AtVSP1* (*AtHAD34*) and *AtVSP2* (*AtHAD33*) also encode Pi starvation-induced secreted acid phosphatases in *Arabidopsis* [52]. These VSP proteins form subgroup IIIc HADs, which have eight and three HAD members in *Arabidopsis* and rice, respectively (Fig 1). Subcellular localization prediction showed that all the subgroup IIIc HADs contained a secreted signal peptide (S2 Table). Similar to *Arabidopsis*, the expression of *OsHAD39* and *OsHAD41* is induced by Pi starvation in roots, indicating that they may be involved in recycling extracellular Po under Pi starvation conditions. In contrast to the subgroup IIIc HAD proteins, other groups of HAD phosphatases do not contain a secreted signal peptide and may be involved in utilizing intercellular Po in plants.

Notably, seven of the 10 subgroup Ⅲa HAD genes were induced by Pi starvation in rice (Figs 6 and 7). These HAD genes belong to the glycerol-3-phosphate acyltransferase (GPAT) family, which participates in the acylation reaction at the *sn*-2 position of glycerol-3-phosphate (G3P) to form *sn*-2 lysophosphatidic acid (*sn*-2 LPA) and further dephosphorylation of *sn*-2 LPA [53, 54]. These GPATs may be involved in recycling Pi from G3P under Pi stress conditions. In *Arabidopsis*, *AtGPAT1~8* (*AtHAD23~28*) are required for the synthesis of cutin and suberin [54]. Interestingly, the expression of *OsHAD27*, *OsHAD29*, *OsHAD33*, and *OsHAD34* was induced more than fivefold after 21 days of Pi starvation in roots (Fig 6). These genes may participate in the synthesis of suberin in roots under Pi-deficient conditions [54].

The cell membrane systems are composed of phospholipids, which are one of the most important Po pools in plants. In *Arabidopsis*, Pi starvation induced the expression of the *AtNPC4* and *AtNPC5* genes to degrade phosphatidyl-choline and phosphatidyl-ethanolamine (PC/PE) to phosphocholine and phosphoethanolamine. *AtPS2* (or *AtHAD1*) and *AtPECP1* (or *AtHAD2*), which belong to the HAD Ia subgroups, further dephosphorylate phosphocholine and phosphoethanolamine to release Pi [22–25]. The gene expression of *OsHAD1* and *OsHAD2*, which are close homologs of *AtHAD1* and *AtHAD2*, was significantly induced under Pi stress conditions, indicating that a similar function of *OsHAD1* and *OsHAD2* may exist in rice. However, mutations in *AtHAD1* and *AtHAD2* do not influence Pi homeostasis or Pi starvation responses in *Arabidopsis*. The functions of *OsHAD1* and *OsHAD2* in Pi starvation adaptations need further analysis.

## Supporting information

S1 Fig Hidden Markov models (HMMs) of HAD proteins from the Pfam database (A) and constructed by reported plant HAD proteins (B).
(PDF)

S2 Fig Sequence search flowchart of the HAD proteins and genes in rice and *Arabidopsis*. (PDF)

S3 Fig Molecular Weight (MW) of the HAD proteins in rice and *Arabidopsis*. (PDF)

S4 Fig Chromosomal location of the HAD genes in rice and *Arabidopsis*. (PDF)

S1 Table Gene information of the HAD superfamily. (XLSX)

S2 Table Secreted signal peptide prediction of HAD family proteins in *Arabidopsis* and rice. (XLSX)

S3 Table Primers used in this study. (XLSX)
