# Supplementary figures and images for: Genome-wide analysis of haloacid dehalogenase genes reveals their function in phosphate starvation responses in rice
Source: PLoS One. 2021 Jan 22;16(1):e0245600. doi: 10.1371/journal.pone.0245600 (PMC7822558; doi:10.1371/journal.pone.0245600)

S1 Fig

(A)

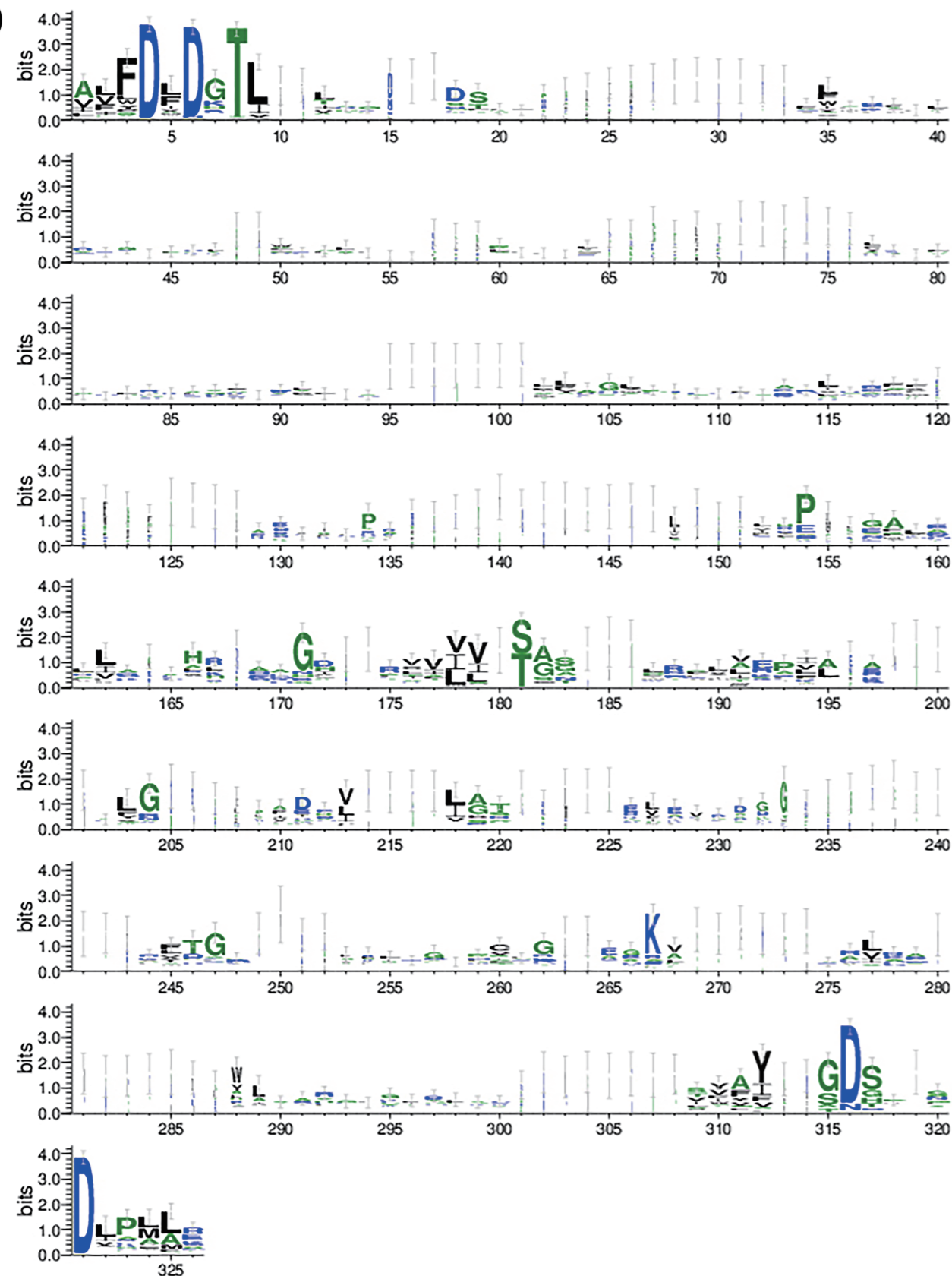

WebLogo 3.6.0

(B)

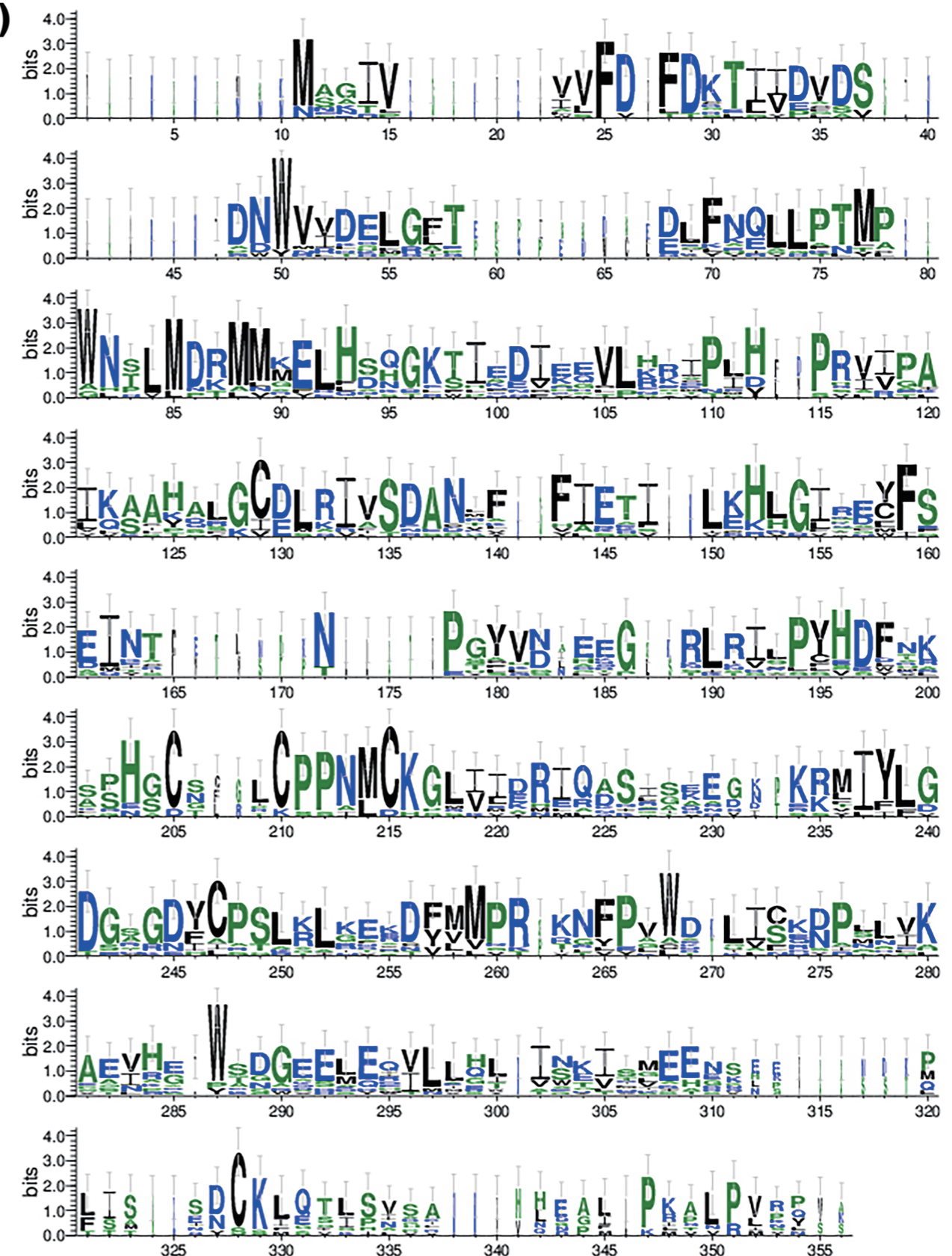

WebLogo 3.6.0

Supplement: S1 Fig — Hidden Markov models (HMMs) of HAD proteins from the Pfam database (A) and constructed by reported plant HAD proteins (B). (PDF) [file pone.0245600.s001.pdf]

**S2 Fig**

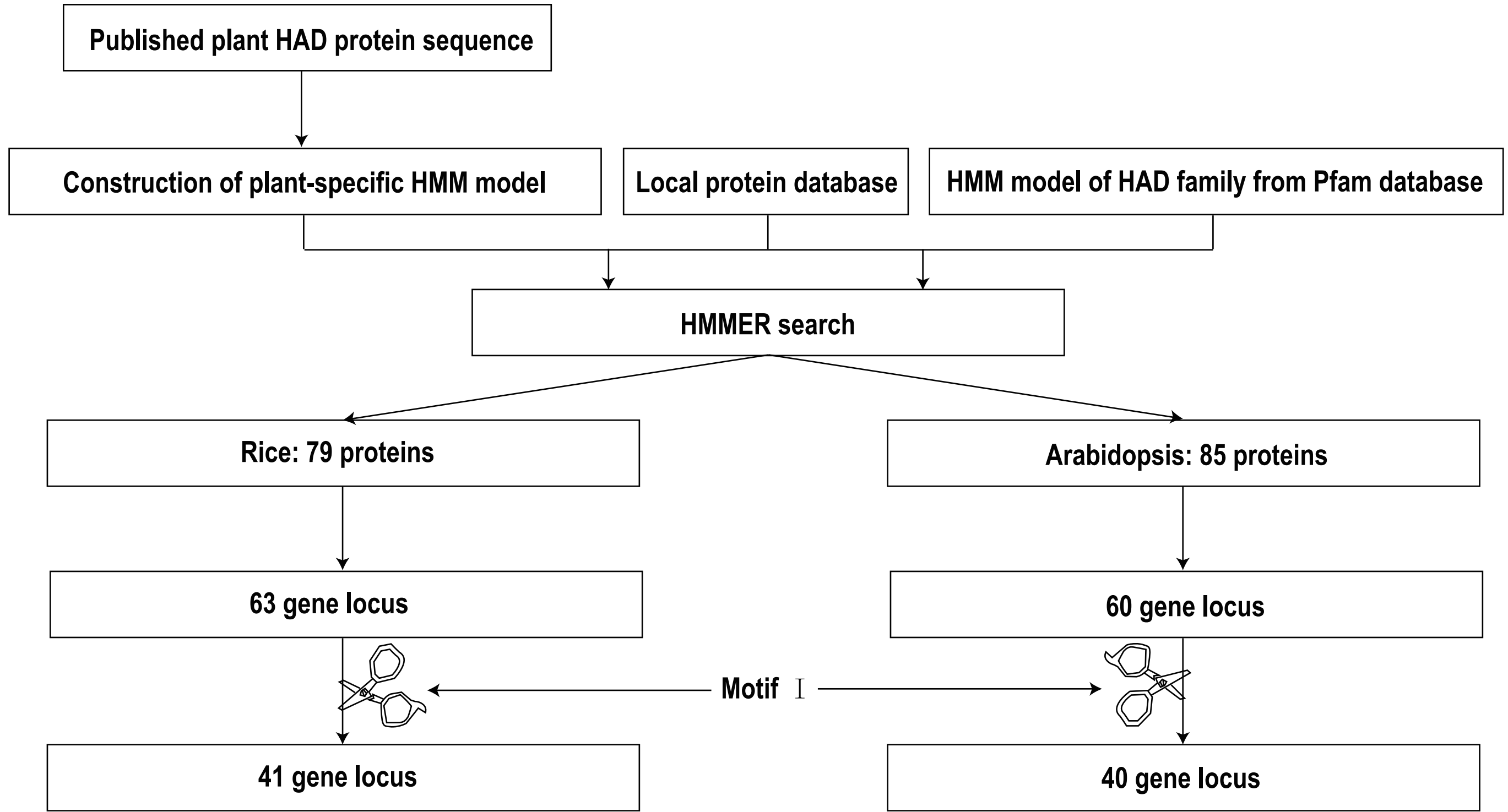

Supplement: S2 Fig — (PDF) [file pone.0245600.s002.pdf]

**S3 Fig**

**Molecular weight (MW)**

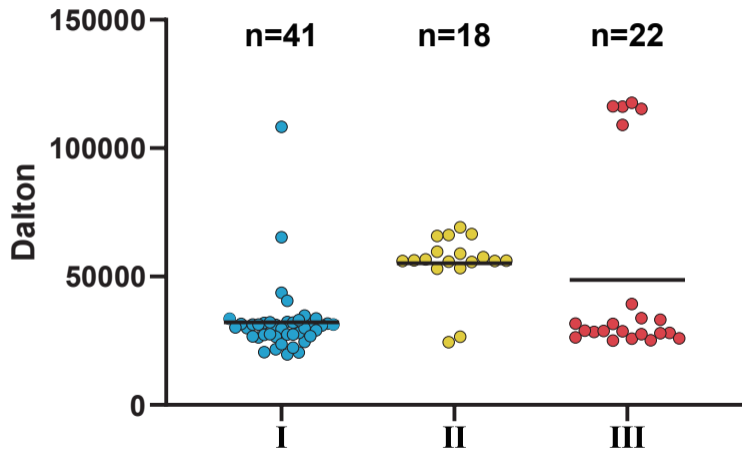

Supplement: S3 Fig — (PDF) [file pone.0245600.s003.pdf]

S4 Fig

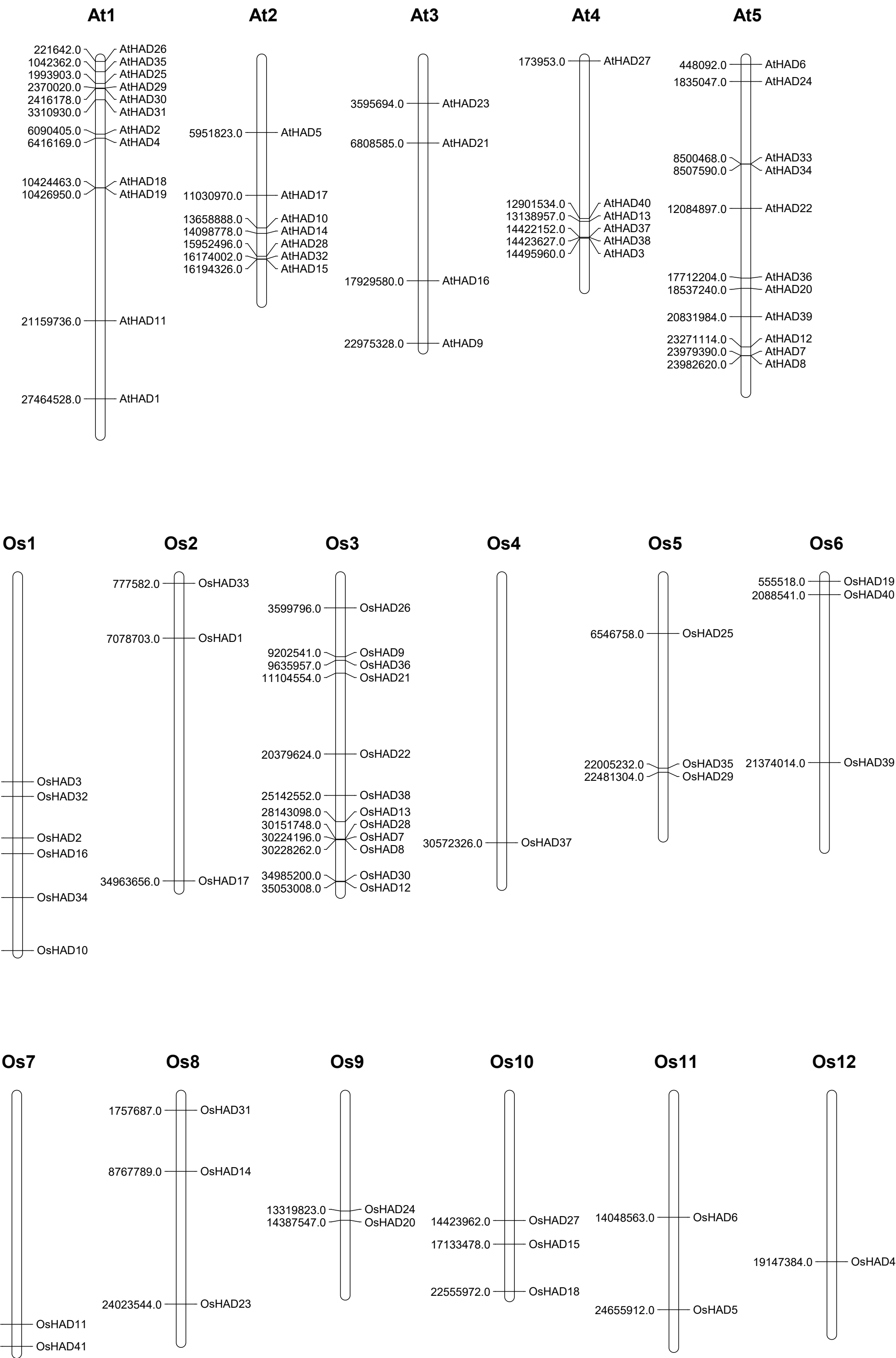

Supplement: S4 Fig — (PDF) [file pone.0245600.s004.pdf]
